# Potential involvement of abnormal splicing in severe *WT1*-related disorders

**DOI:** 10.1007/s10157-025-02715-7

**Published:** 2025-06-10

**Authors:** China Nagano, Masafumi Matuso, Yuta Inoki, Yu Tanaka, Yuta Ichikawa, Chika Ueda, Hideaki Kitakado, Nana Sakakibara, Tomoko Horinouchi, Tomohiko Yamamura, Shingo Ishimori, Kandai Nozu

**Affiliations:** 1 Department of Pediatrics, Kobe University Graduate School of Medicine, 7-5-2 Kusunoki-cho, Chuo-ku, Kobe, Hyogo 650-0017 Japan; 2 Graduate School of Science, Technology and Innovation, Kobe University, 1-1 Rokkodai-cho, Nada-ku, Kobe, Hyogo 657-8501 Japan

**Keywords:** WT1, Splicing, Severity, Genotype–phenotype

## Abstract

**Introduction:**

*WT1*-related disorders are associated with *WT1* gene variants. Recent advances in genetic medicine have led to a better understanding of the genotype–phenotype correlation in *WT1*-related diseases, particularly missense variants in exons 8 or 9 that lead to a wide range of severities. Exonic variants can lead to splicing abnormalities in rare diseases. No reports have investigated exonic variants in *WT1* that cause aberrant splicing. We examined whether exonic variants in *WT1* exon 8 or 9 cause splicing abnormalities and affect disease severity.

**Methods:**

We selected nine rare missense variants in exon 8 or 9 in *WT1* outside the DNA binding domain and C2H2 sites from the Human Gene Variant Database Professional that unexpectedly present severe phenotypes. We conducted functional splicing assays using hybrid minigenes for the nine variants containing exon 8 and 9 and surrounding sequences. Minigene vectors were transfected into cultured cells, and mRNA was analyzed. In silico analysis was performed.

**Results:**

Splicing assays revealed that one of the nine variants caused aberrant splicing, with exon 8 skipping. One previously reported case with this variant showed particularly severe phenotype, progressing to kidney failure within 3 months.

**Conclusions:**

One *WT1* variant in exon 8 or 9 disrupted the splice site, leading to aberrant splicing in vitro and potentially contributing to an unexpectedly severe phenotype for a missense variant outside the DNA binding domain and C2H2 sites. In vitro splicing assays may help clarify the genotype–phenotype correlation in *WT1*-related disorders, especially for variants outside canonical functional domains.

**Supplementary Information:**

The online version contains supplementary material available at 10.1007/s10157-025-02715-7.

## Introduction

*WT1*-related disorders are caused by heterozygous variants in the *WT1* gene [1]. These disorders include various syndromes, such as Denys–Drash syndrome (OMIM 194070), Frasier syndrome (OMIM 136680), Meacham syndrome (OMIM 608978), and WAGR syndrome (Wilms tumor, aniridia, urogenital malformations, and various mental retardations, OMIM 194072). A genotype–phenotype correlation has been established for *WT1*-related disorders; the location and type of *WT1* variants significantly influence the clinical presentation, severity of the condition, and the chronology of patient manifestations [2, 3]. The WT1 protein has four zinc finger structures (DNA binding domain) at the C-terminus, which bind to transcriptional regulatory sequences on DNA, and acts as a transcription factor. The four C2H2–zinc fingers correspond to exons 7–10, respectively.

Patients with missense variants of exons 8 or 9, which correspond to the DNA binding domain, show severe symptoms. We previously evaluated the genotype–phenotype correlations in a systematic review of 174 cases with *WT1* exon 8 to 9 variants [4]. We classified the variants into three categories by the location of the mutation in the WT1 protein: DNA binding site variant, C2H2 site variant, and variants in other sites. Among the 174 variants, 95 were DNA binding site variants, 38 were C2H2 site variants, and 41 were other site variants. The median age of developing kidney failure was 0.90 years in the DNA binding site group, 2.00 years in the C2H2 site group, and 3.92 years in the other site group [5]. We concluded that the DNA binding and C2H2 zinc finger structures are important for maintaining WT1 transcriptional activity, and variants in these domains causes severe clinical symptoms.

RNA splicing is a process that involves the removal of intervening, noncoding sequences of genes (introns) from pre-mRNA and the joining of the protein-coding sequences (exons) to enable translation of mRNA into a protein. Missense, nonsense, and synonymous variants within coding exons, as well as intronic variants, can disrupt pre-mRNA splicing and cause disease [6, 7]. Given the growing evidence that splicing aberrations frequently contribute to rare disease etiology, careful clinical interpretation of variants may lead to better understanding of disease mechanisms.

Patients with *WT1* variants in exons 8 or 9 at the other sites are expected to show milder phenotypes but, in fact, show a range of severity. Against this background, this study investigated the potential involvement of splicing abnormalities in clinically severe cases with variants in the *WT1* gene outside the DNA binding domain and C2H2 sites.

## Methods

### Patients and variants

Candidate variants for this study were selected from the Human Gene Variant Database (HGMD) Professional. We previously reviewed 174 cases that carried *WT1* (NM_024426) exon 8 or 9 missense variants [4]. Among these 174 patients, 41 had variants outside the DNA binding domain and C2H2 sites in the *WT1* gene and were expected to show mild phenotypes of later onset of kidney failure. Of the 41 cases, nine fit the definition of clinically relatively severe as cases that led to kidney failure at younger than 5 years of age. The variants are shown in Table 1.

**Table 1:** In vitro (Minigene) and in silico assays

	Exon	Gene variants (NM_024426)	In vitro (minigene assay)	In silico (SpliceAI)							
			Result	Acceptor Loss		Donor Loss		Acceptor Gain		Donor Gain	
				Δscore	Position	Δscore	Position	Δscore	Position	Δscore	Position
No. 1	8	c.1304G > C p.(Arg435Pro)	Normal	0.04	− 652 bp	0.05	− 744 bp	0		0	
No. 2	8	c.1309 T > C p.(Phe437Leu)	Normal	0	− 13 bp	0	2034 bp	0.01	44 bp	0.01	−739 bp
No. 3	8	c.1352C > G p.(Thr451Arg)	Normal	0.09	87 bp	0.08	− 2 bp	0		0.02	−6 bp
No. 4	8	c.1354G > T p.(Gly452Cys)	Exon 8 skipping	0.57	89 bp	0.98	0 bp	0		0.25	−4 bp
No. 5	9	c.1366 T > C p.(Phe456Leu)	Normal	0.01	− 6 bp	0	− 1677 bp	0.02	11 bp	0.01	−81 bp
No. 6	9	c.1405G > A p.(Asp469Asn)	Normal	0.03	50 bp	0.03	− 42 bp	0		0	−1638 bp
No. 7	9	c.1405G > C p.(Asp469His)	Normal	0.08	50 bp	0.09	−42 bp	0		0	−1638 bp
No. 8	9	c.1405G > T p.(Asp469Tyr)	Normal	0.04	50 bp	0.04	− 42 bp	0		0	−1638 bp
No. 9	9	c.1406A > G p.(Asp469Gly)	Normal	0.03	51 bp	0.04	− 41 bp	0		0	−1637 bp

### In vitro* assay*

To create hybrid minigene constructs, we used the H492v vector based on the pcDNA 3.0 mammalian expression vector (Invitrogen, Carlsbad, CA, USA) that we previously developed (Supplemental Figure S1) [8–13]. We cloned DNA fragments containing exons 8 and 9 and the surrounding introns of the *WT1* gene into the H492v vector using In-Fusion cloning methods with the In-Fusion^®^ HD Cloning Kit (Takara Bio Inc., Kusatsu, Japan), in accordance with the manufacturer’s instructions.

Because gDNA of patients was not available, we initiated cloning from wild-type gDNA and then introduced mutations by site-directed mutagenesis using the PrimeSTAR^®^ Mutagenesis Basal Kit (Takara Bio Inc.), in accordance with the manufacturer’s instructions. The primers are shown in Supplemental Table S1.

**Fig. 1:**
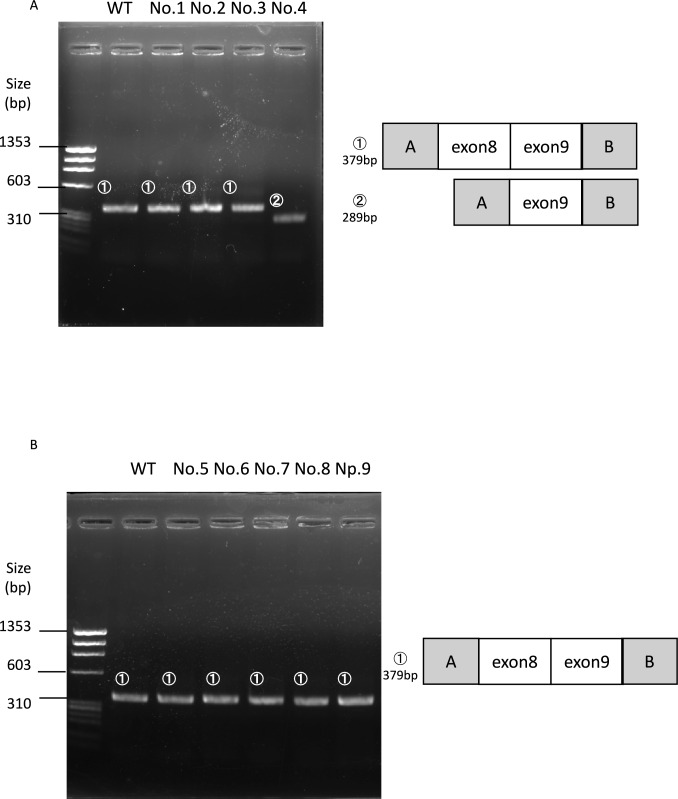
Electrophoresis results from the minigene constructs. The sequences are shown in Supplementary Fig. 2. The schematic on the right shows the wild-type sequence and sequence with exon 8 skipping. **A** Wild type (WT), No. 1, No. 2, and No. 3 exhibited a single band (379 bp) corresponding to exon 8 and 9 in *WT1* and No. 4 exhibited a smaller band (289 bp) corresponding to exon 8 skipping. **B** Wild type (WT), No. 5, No. 6, No. 7, No. 8, and No. 9 exhibited a single band (379 bp) corresponding to exon 8 and 9 in *WT1*

The hybrid minigenes were confirmed by sequencing and transfected into HEK293T cells using the Lipofectamine® 3000 Transfection Kit (Thermo Fisher Scientific, Waltham, MA, USA). Total RNA was extracted from cells after 24 h using the RNeasy® Plus Mini Kit (QIAGEN, Hilden, Germany). Total RNA (1 µg) was reverse-transcribed to cDNA with EcoDry™ Premix (Double Primed) (Takara Bio Inc.). PCR was performed using a forward primer corresponding to a sequence upstream of exon A (from the H492v vector) and a reverse primer complementary to a sequence downstream of exon B (from the H492v vector) (Supplemental Figure S1, Fig. 1). PCR products were analyzed by electrophoresis on a 1.5% agarose gel using a φX 174-Hae III digest DNA ladder and direct sequencing.

### In silico* assay*

We predicted the splicing domain strength in each variant using Splice AI (https://spliceailookup.broadinstitute.org/). Scores obtained using Splice AI are shown in Table 1.

## Results

### Minigene assay

We evaluated all nine variants using the minigene splicing assay. Among them, only one variant, c.1354G > T (Gly452Cys), exhibited exon 8 skipping, as evidenced by a shorter RT-PCR product (289 bp) compared to the wild-type transcript (379 bp). This skipping was confirmed by sequencing (Fig. 1, Supplemental Fig. S2). The other eight variants, including c.1304G > C, c.1309 T > C, and c.1406A > G, showed normal splicing patterns identical to the wild type.

### In silico* assay*

We examined the change of splicing score for each gene variant using Splice AI. We specified the distance for 10,000 bp. In silico analysis using SpliceAI showed a high donor loss score (0.98) for the Gly452Cys variant, consistent with exon 8 skipping. In contrast, the other eight variants showed negligible scores (≤ 0.09 for donor/acceptor loss), supporting the observed minigene results (Table 1). Variant-specific details are provided in Table 1.

#### Clinical correlation

Clinically, the Gly452Cys variant has been reported in one patient who progressed to kidney failure within the first 3 months of life (Table 2, Supplemental Table S2). This early onset is comparable to that observed in DNA-binding domain variants, highlighting the potential impact of exon skipping on *WT1* function.

**Table 2:** Clinical characteristics of patients with nine *WT1* exon 8/9 variants

No	Variant (cDNA/protein)	Age at Onset (y)	Age at Kidney Failure (y)	Wilms’ Tumor	Cases (ID) in Sup Table S2
1	c.1304G > C/p.Arg435Pro	1.75	1.75	No	Case 1
2	c.1309 T > C/p.Phe437Leu	0.5	0.75	No	Case 2
3	c.1352C > G/p.Thr451Arg	1.3, 5.0	1.3, NA	Yes, NA	Cases 3, 4
4	c.1354G > T/p.Gly452Cys	0.25	0.25	No	Case 5
5	c.1366 T > C/p.Phe456Leu	0.67	3.0	No	Case 6
6	c.1405G > A/p.Asp469Asn	0.08–1.58	0.33–3.92, some NA	Mixed	Cases 7–17
7	c.1405G > C/p.Asp469His	0.25	0.33	Yes	Case 18
8	c.1405G > T/p.Asp469Tyr	0.02, 0.08	Died at 6 m, 0.08	NA	Cases 19, 20
9	c.1406A > G/p.Asp469Gly	0.33	1.0	Yes	Case 21

^*^NA not available

## Discussion

Research has shown that some variants can disrupt original splice sites, affect the splicing pattern, and influence disease severity. This is the first report examining the possibilities of splicing abnormalities caused by single base substitutions that were expected to be missense variants of the *WT1* gene.

We previously reviewed 174 cases with *WT1* exon 8 and 9 missense variants and found that the median age of developing kidney failure was 0.90 in the DNA binding site group, 2.00 in the C2H2 site group, and 3.92 years in the other site group [5]. In this study, we focused on the 41 cases in the other site group. Most showed mild phenotypes, but some cases showed unexpectedly severe phenotypes. To explore the reason for the severe phenotype, we selected variants in patients with kidney failure before the age of 5 years and examined the possibility of splicing abnormalities caused by these single-base substitutions. These nine variants were specifically selected from patients with early onset kidney failure. Therefore, by study design, the same variants were not expected to occur in milder cases in our cohort. Our results showed that one case, No. 4, harbored a splicing abnormality in the *WT1* gene. The splicing anomaly in No. 4 caused exon 8 skipping, which is an in-frame variant, because exon 8 is a multiple of 3. Although this variant is not located in the DNA binding site, we hypothesize that the exon 8 skipping leads to production of an in-frame truncated WT1 protein. While this may impair DNA binding, further studies are needed to assess its dominant-negative potential and transcriptional impact. One reported patients with this variant showed kidney failure within 3 months (Supplemental Table S2, No. 5) and appear to have the same severity as patients with variants in the DNA binding site [14, 15]. In contrast to the well-characterized KTS/− KTS isoform imbalance observed in Frasier syndrome, which results from splicing alterations in intron 9, the exon 8 skipping identified in this study leads to complete exclusion of a protein-coding exon. While both mechanisms involve aberrant splicing, their consequences differ fundamentally. Frasier syndrome is caused by altered ratios of WT1 isoforms (with and without the KTS insert), which modulate target gene expression patterns. By comparison, exon 8 skipping eliminates a structurally important exon, potentially impairing DNA-binding capacity more directly.

In *WT1*-related disorders, the mutant WT1 protein is thought to dimerize with the wild-type protein and act in a dominant-negative manner, resulting in a more severe phenotype with missense variants compared with haploinsufficiency [16]. So far, only two in-frame mutations in exons 8 or 9 have been reported (c.1342_1347delAGGAGA and c.1419_1430del12) [17, 18]. The age of onset of these cases was at birth and 0.1 years, and the age of kidney failure was very early in both cases, at birth and 1.1 years. While the variant positions in both cases were not in the DNA binding site, it is assumed that the in-frame deletion disrupted the WT1 structure but resulted in the production of an abnormal protein, leading to reduced DNA binding ability. Thus, in-frame variants in exon 8 or 9 may result in the expression of WT1 with reduced DNA binding ability similar to that of DNA binding site missense variants, leading to severe phenotypes.

Variants in splicing acceptor and donor sites (AG–GT sites) alter the interaction between the pre-mRNA and the mRNA involved in intron removal. In recent years, several reports have shown that variants within exons, as well as variants in acceptor and donor sites, can lead to splicing abnormalities in patients with rare diseases [19–21]. Notably, these variants are usually missense variants. Even though these variants occur within coding exons, they can disrupt splicing by influencing splice sites or interacting with splicing regulatory elements, such as exonic splicing enhancers and silencers. The variant in No. 4 in this study is located at the last nucleotide position in exon 8. We previously showed that a single-base substitution at the last nucleotide position in each exon in *COL4A5* resulted in a high rate of splicing abnormalities, because single base substitutions lead to a weaker 5′ splice site, which reduces the removal of the upstream intron [22]. The same may be for the *WT1* gene. Variants in the second or third base from the end of exons in *COL4A5* have also been shown to be responsible for splicing abnormalities [20]. Thus, close attention is required when interpreting exonic single nucleotide variants near the 5′ splice site.

Of the nine variants analyzed, eight exhibited no splicing abnormalities yet were associated with clinically severe phenotypes. The underlying mechanism contributing to the severity of these variants may involve modifier genes or epigenetic regulation. These variants may influence WT1 protein structure, stability, or interactions independent of splicing, potentially disrupting DNA binding or transcriptional regulation. Although our splicing assay found no defects, functional consequences beyond splicing must be considered to explain disease severity in these cases. Given that WT1 functions as a transcription factor, it is of significant interest to investigate its potential involvement in DNA methylation and histone modification, both of which play critical roles in gene expression. With recent advancements in epigenomics research, further exploration in this area is highly warranted to elucidate these mechanisms.

Phenotypic differences between variants of rare diseases can result from several mechanisms, which may involve genetic, epigenetic, environmental, or other mechanisms. These mechanisms explain why individuals with variants in the same gene might exhibit varying disease presentations. The present study focused on differences in splicing as a factor that influences the severity of disease, and further research is needed to determine whether other mechanisms are involved in the range of disease severity in this patient group. A major limitation is that splicing effects were validated only in vitro. Ideally, confirmation using RNA from patient-derived tissues (e.g., kidney biopsy or blood lymphocytes) is needed.

In conclusion, this study suggests that abnormal splicing caused by a specific *WT1* exon 8 variant may contribute to the severe phenotype observed in affected individuals. This finding was derived from minigene assays and supported by in silico prediction. However, several other missense variants without splicing alterations were also associated with severe disease, indicating that alternative pathogenic mechanisms such as impaired protein function or dominant-negative effects may play a role. These findings underscore the importance of functional assays and cautious interpretation when evaluating *WT1* variants outside the DNA-binding domains.

## Supplementary Information

Below is the link to the electronic supplementary material.

Supplementary file1 (DOCX 6089 KB)

## Data Availability

The data sets generated and/or analyzed during the current study are available from the corresponding author on reasonable request.
