# Supplementary material for: Potential involvement of abnormal splicing in severe WT1-related disorders
Source: Clin Exp Nephrol. 2025 Jun 10;29(10):1489–95. doi: 10.1007/s10157-025-02715-7 (PMC12464044; doi:10.1007/s10157-025-02715-7)
Supplement: Supplementary file 1 — Supplementary file1 (DOCX 6089 KB) [file 10157_2025_2715_MOESM1_ESM.docx]

**Supplemental Material Table of Contents**

Supplemental Table S1. Primer sequences

Supplemental Table S2. Characteristics of 21 cases with variants outside the DNA binding domain and C2H2 sites in the *WT1* gene who showed severe symptoms

Supplemental Figure S1. Schematic representation of the H492v vector

Supplemental Figure S2. Sequences of RT-PCR product generated from minigene assay

Supplemental Table S1: The primers for cloning and mutagenesis

|  | | 5’ 3’ |
| --- | --- | --- |
| Exon 8, 9 | F | CCGTGCTTTGTTAGC TCCTACCCTAACAAGCTCCAG |
|  | R | TCGATGTTAACGCTA AGCCACGCACTATTCCTTC |
| No. 1 | F | TGTGAACCAAGGTTTTCTCGTTCAGA |
|  | R | AAACCTTGGTTCACAGTCCTTGAAGTC |
| No. 2 | F | ACGAAGGCTTTCTCGTTCAGACCAGC |
|  | R | CGAGAAAGCCTTCGTTCACAGTCCTTG |
| No. 3 | F | AGACATAGAGGTTTGTAGGTTCACTTC |
|  | R | CAAACCTCTATGTCTCCTTTGGTGTC |
| No. 4 | F | ACATACATGTTTGTAGGTTCACTTCTC |
|  | R | TACAAACATGTATGTCTCCTTTGGTG |
| No. 5 | F | AAACCATCCCAGTGTAAAACTTGTCA |
|  | R | ACACTGGGATGGTTTCACACCTGTA |
| No. 6 | F | CCGGTCCAACCACCTGAAGACCCACA |
|  | R | AGGTGGTTGGACCGGGAGAACTTTCG |
| No. 7 | F | CCGGTCCCACCACCTGAAGACCCACA |
|  | R | AGGTGGTGGGACCGGGAGAACTTTCG |
| No. 8 | F | CCGGTCCTACCACCTGAAGACCCACA |
|  | R | AGGTGGTAGGACCGGGAGAACTTTCG |
| No. 9 | F | CGGTCCGGCCACCTGAAGACCCACAC |
|  | R | CAGGTGGCCGGACCGGGAGAACTTTC |
| H492v  plasmid | F | ATTACTCGCTCAGAAGCTGTGTTGC |
|  | R | AAGTCTCTCACTTAGCAACTGGCAG |

Supplemental Table S2. Characteristics of 21 cases with variants outside the DNA binding domain and C2H2 sites in the *WT1* gene who showed severe symptoms

| No. | Sex | Age at onset (y) | Age at kidney failure (y) | Extra- renal symptom | Wilms' tumor | Exon | Gene variants | | reference |
| --- | --- | --- | --- | --- | --- | --- | --- | --- | --- |
| 1 | M | 1.75 | 1.75 |  | No | 8 | 1304G>C | Arg435Pro | 1 |
| 2 | M | 0.50 | 0.75 |  | No | 8 | 1309T>C | Phe437Leu | 2 |
| 3 | M | 1.30 | 1.30 | No | Yes | 8 | 1352C>G | Thr451Arg | 3 |
| 4 | F | 5.00 |  |  |  | 8 | 1352C>G | Thr451Arg | 4 |
| 5 | F | 0.25 | 0.25 |  | No | 9 | 1354G>T | Gly452Cys | 2 |
| 6 | M | 0.67 | 3.00 |  | No | 9 | 1366T>C | Phe456Leu | 5 |
| 7 | M | 0.83 | no | No | No | 9 | 1405G>A | Asp469Asn | 6 |
| 8 | M | 0.25 | 1.42 |  | No | 9 | 1405G>A | Asp469Asn | 7 |
| 9 | F | 1.58 | 3.92 |  | Yes | 9 | 1405G>A | Asp469Asn | 7 |
| 10 | M | 0.33 | 0.33 |  | No | 9 | 1405G>A | Asp469Asn | 7 |
| 11 | F | 1.10 | 2.50 |  | Yes | 9 | 1405G>A | Asp469Asn | 7 |
| 12 | F | 1.50 | no (at 5) |  | Yes | 9 | 1405G>A | Asp469Asn | 3 |
| 13 | M | 0.64 | 0.64 | No | No | 9 | 1405G>A | Asp469Asn | 2 |
| 14 | F | 1.00 | 2.25 |  | No | 9 | 1405G>A | Asp469Asn | 5 |
| 15 | F | 0.17 |  |  |  | 9 | 1405G>A | Asp469Asn | 8 |
| 16 | M | 1.17 | 1.17 | No | No | 9 | 1405G>A | Asp469Asn | 9 |
| 17 | F | 0.08 |  |  |  | 9 | 1405G>A | Asp469Asn | 4 |
| 18 | M | 0.25 | 0.33 |  | Yes | 9 | 1405G>C | Asp469His | 10 |
| 19 | F | 0.02 | died at 6 mo |  |  | 9 | 1405G>T | Asp469Tyr | 11 |
| 20 | F | 0.08 | 0.08 |  |  | 9 | 1405G>T | Asp469Tyr | 12 |
| 21 | F | 0.33 | 1.00 |  | Yes | 9 | 1406A>G | Asp469Gly | 7 |

Reference

1. Lehnhardt A, Karnatz C, Ahlenstiel-Grunow T, Benz K, Benz MR, Budde K, et al.: Clinical and molecular characterization of patients with heterozygous mutations in wilms tumor suppressor gene 1. Clinical journal of the American Society of Nephrology : CJASN,10: 825-831, 2015 10.2215/CJN.10141014

2. Schumacher V, Scharer K, Wuhl E, Altrogge H, Bonzel KE, Guschmann M, et al.: Spectrum of early onset nephrotic syndrome associated with WT1 missense mutations. Kidney international,53: 1594-1600, 1998 10.1046/j.1523-1755.1998.00948.x

3. Chernin G, Vega-Warner V, Schoeb DS, Heeringa SF, Ovunc B, Saisawat P, et al.: Genotype/phenotype correlation in nephrotic syndrome caused by WT1 mutations. Clinical journal of the American Society of Nephrology : CJASN,5: 1655-1662, 2010 10.2215/CJN.09351209

4. Sadowski CE, Lovric S, Ashraf S, Pabst WL, Gee HY, Kohl S, et al.: A single-gene cause in 29.5% of cases of steroid-resistant nephrotic syndrome. Journal of the American Society of Nephrology : JASN,26: 1279-1289, 2015 10.1681/ASN.2014050489

5. Jeanpierre C, Denamur E, Henry I, Cabanis MO, Luce S, Cecille A, et al.: Identification of constitutional WT1 mutations, in patients with isolated diffuse mesangial sclerosis, and analysis of genotype/phenotype correlations by use of a computerized mutation database. American journal of human genetics,62: 824-833, 1998

10.1086/301806

6. Bezdicka M, Stolbova S, Seeman T, Cinek O, Malina M, Simankova N, et al.: Genetic diagnosis of steroid-resistant nephrotic syndrome in a longitudinal collection of Czech and Slovak patients: a high proportion of causative variants in NUP93. Pediatric nephrology,33: 1347-1363, 2018 10.1007/s00467-018-3950-2

7. Hohenstein P, Hastie ND: The many facets of the Wilms' tumour gene, WT1. Human molecular genetics,15 Spec No 2: R196-201, 2006 10.1093/hmg/ddl196

8. Gbadegesin R, Hinkes BG, Hoskins BE, Vlangos CN, Heeringa SF, Liu J, et al.: Mutations in PLCE1 are a major cause of isolated diffuse mesangial sclerosis (IDMS). Nephrology, dialysis, transplantation : official publication of the European Dialysis and Transplant Association -European Renal Association,23: 1291-1297, 2008 10.1093/ndt/gfm759

9. Takata A, Kikuchi H, Fukuzawa R, Ito S, Honda M, Hata J: Constitutional WT1 correlate with clinical features in children with progressive nephropathy. Journal of medical genetics,37: 698-701, 2000 10.1136/jmg.37.9.698

10. Pritchard-Jones K, Fleming S, Davidson D, Bickmore W, Porteous D, Gosden C, et al.: The candidate Wilms' tumour gene is involved in genitourinary development. Nature,346: 194-197, 1990 10.1038/346194a0

11. Lee JH, Han KH, Lee H, Kang HG, Moon KC, Shin JI, et al.: Genetic basis of congenital and infantile nephrotic syndromes. American journal of kidney diseases : the official journal of the National Kidney Foundation,58: 1042-1043, 2011 10.1053/j.ajkd.2011.09.007

12. Hahn H, Cho YM, Park YS, You HW, Cheong HI: Two cases of isolated diffuse mesangial sclerosis with WT1 mutations. Journal of Korean medical science,21: 160-164, 2006 10.3346/jkms.2006.21.1.160

**Supplemental Figure S1.** Schematic representation of the H492v vector


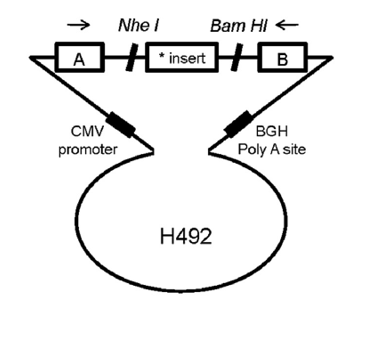


**H492v**

**Supplemental Figure S2.** Sequences of RT-PCR product generated from minigene assay

**(A)**  WT full


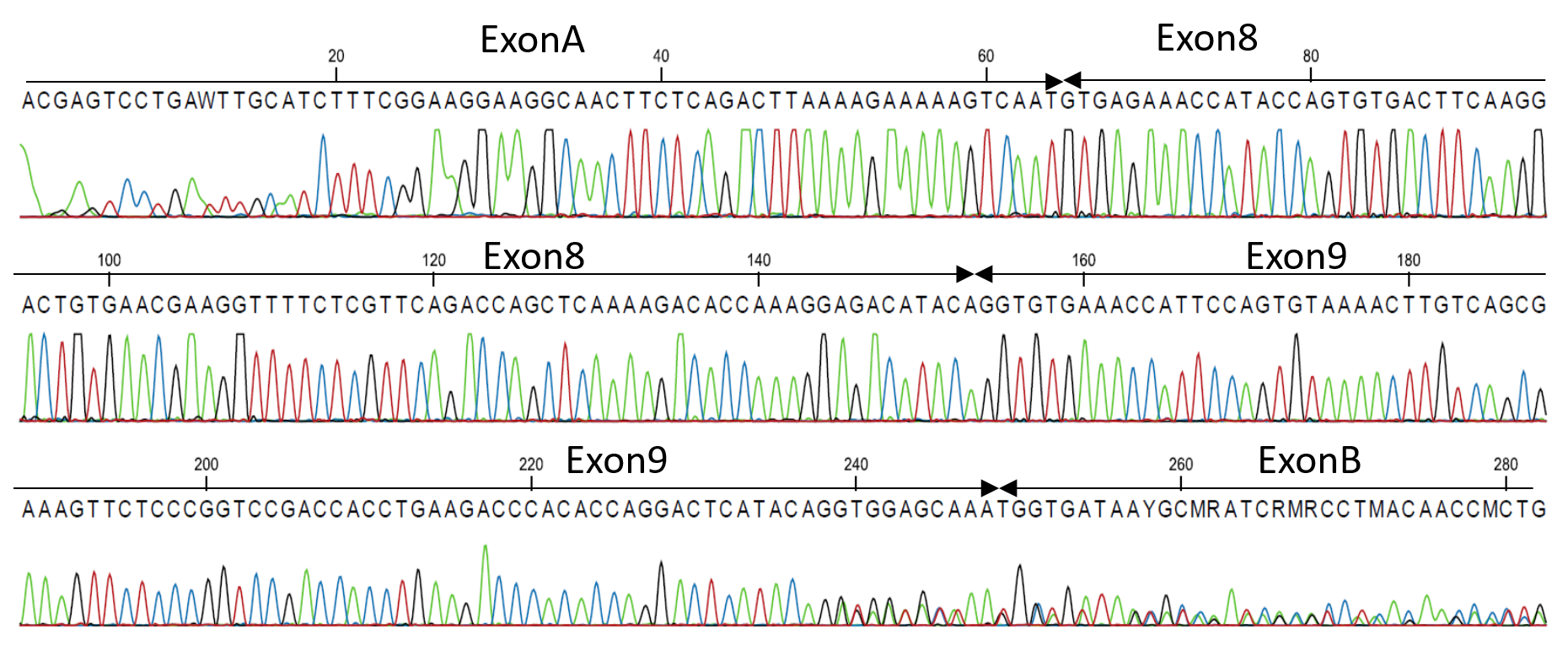


(B)　No. 1, c.1304G>C


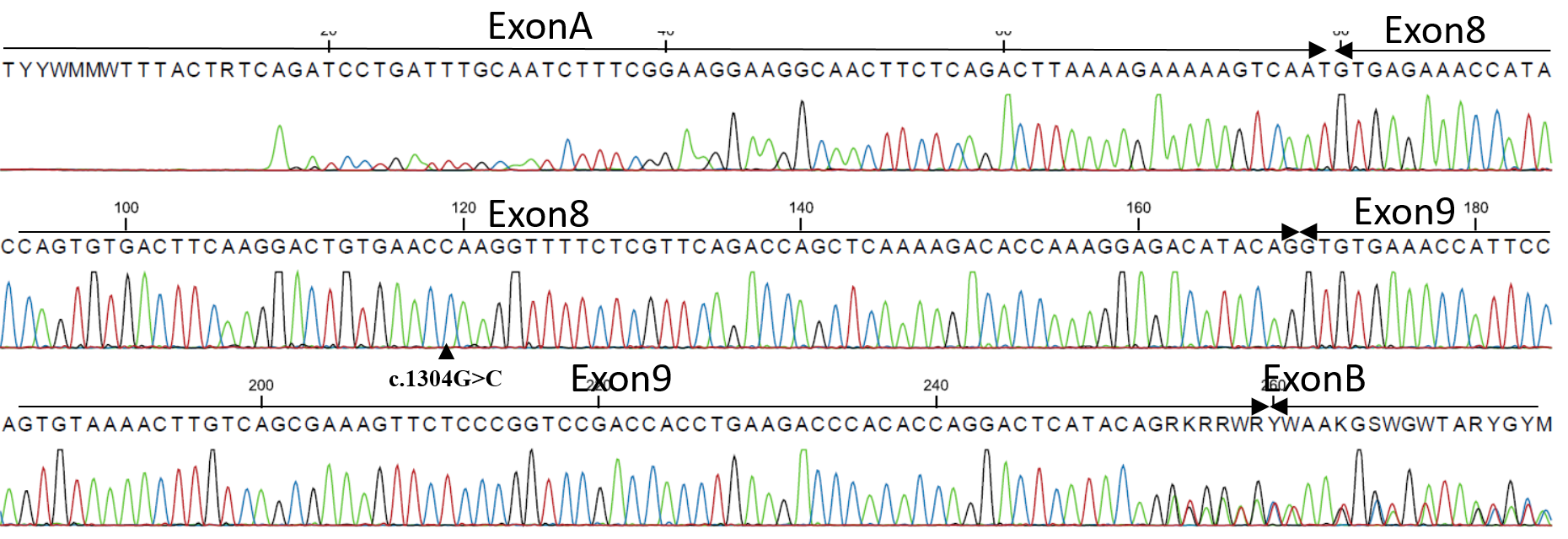


(C) No. 2, c.1309T>C


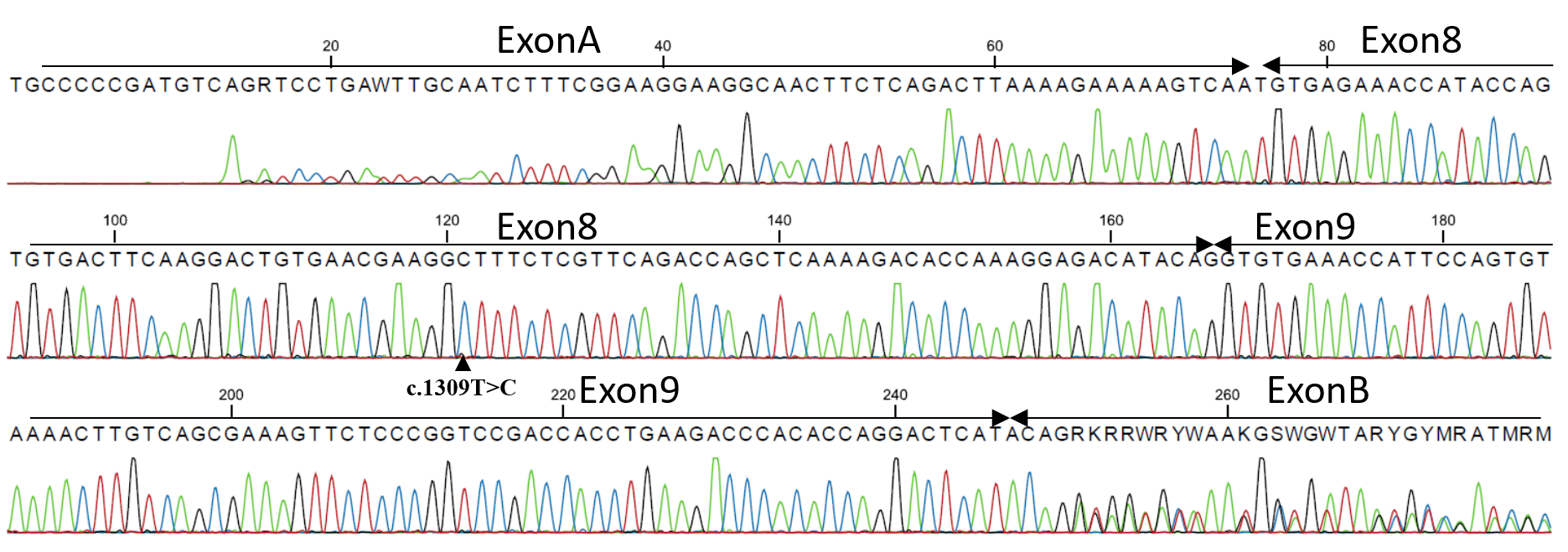


(D) No. 3, c.1352C>G


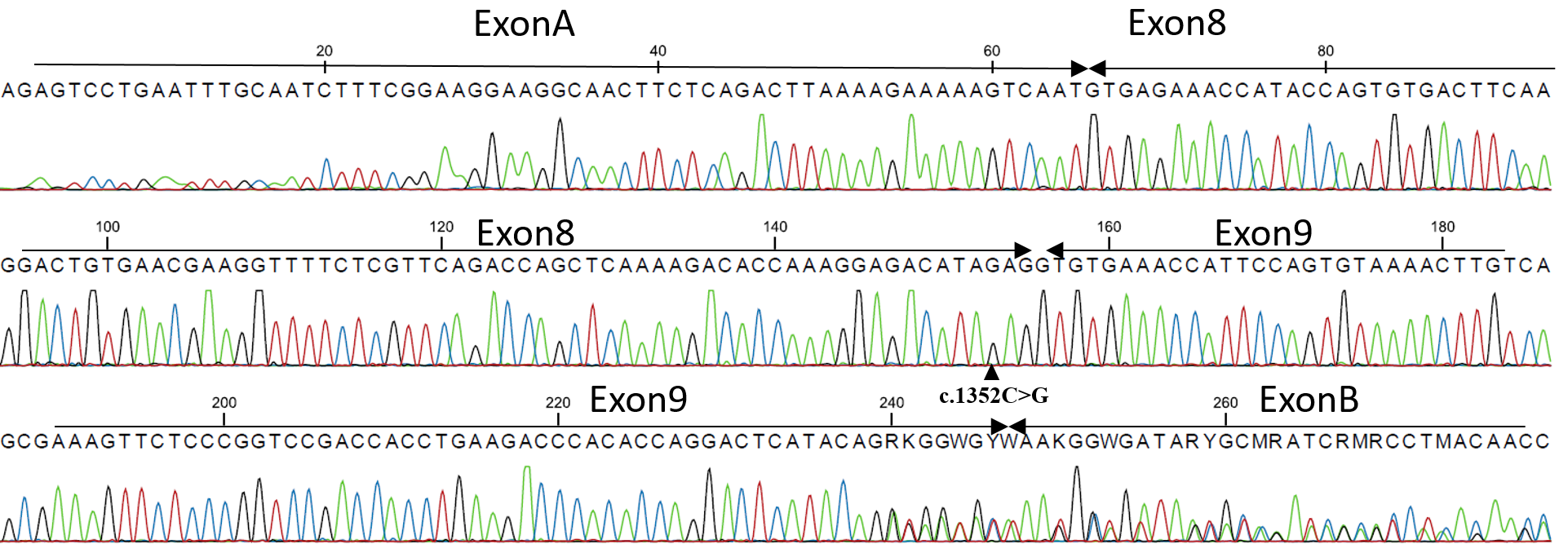


(E)　No.4, c.1354G>T


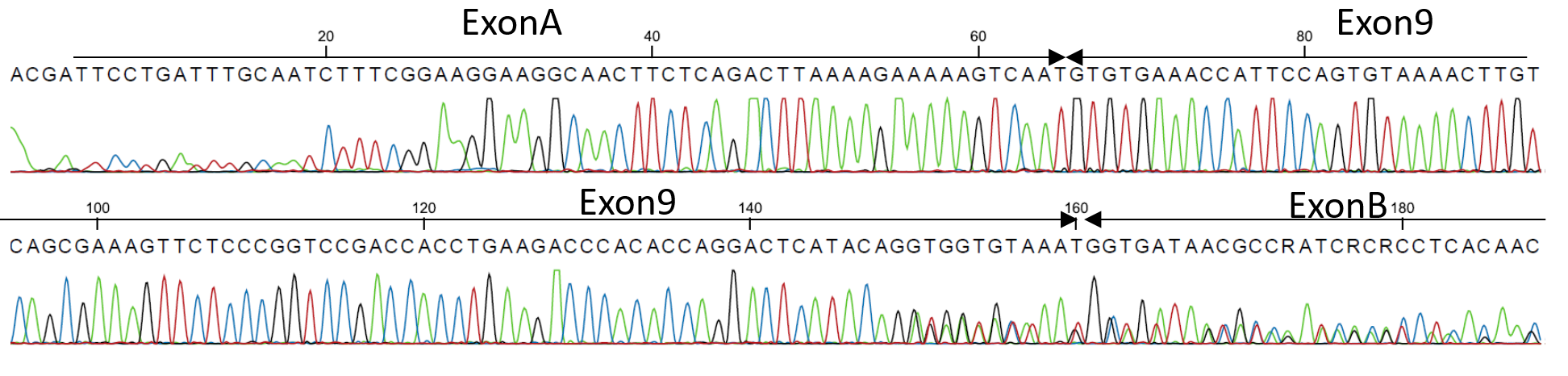


(F)　No.5, c.1366T>C


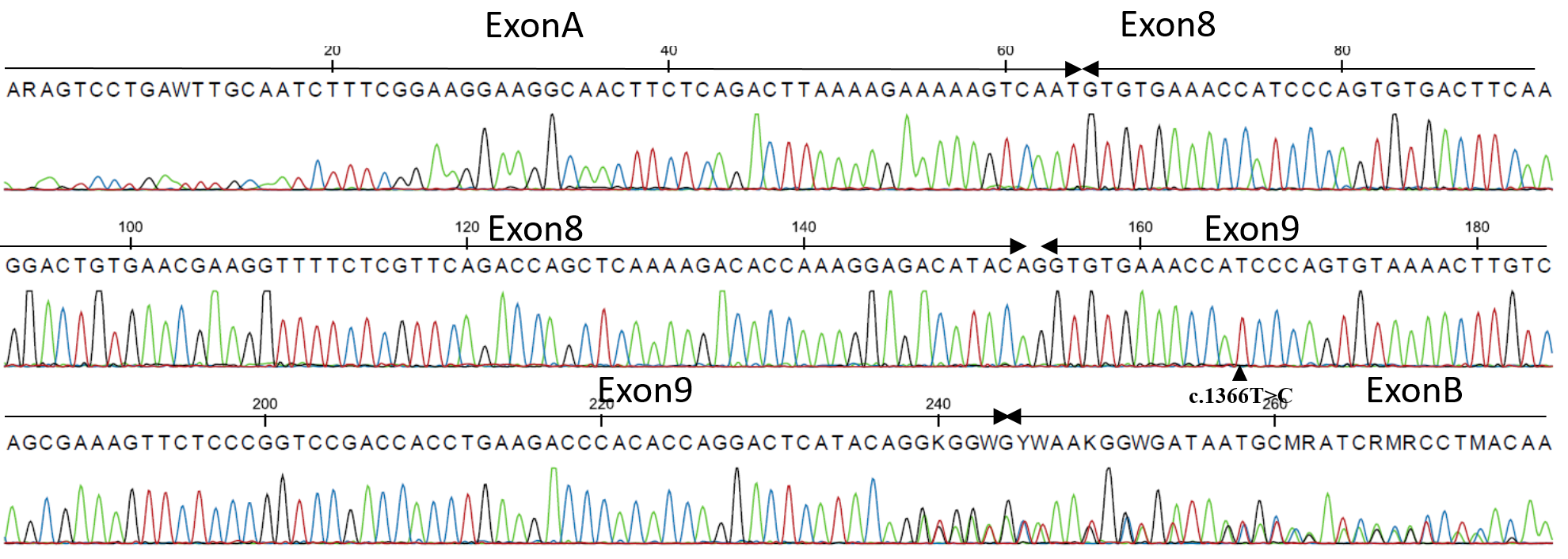


(G)　No. 6, c.1405G>A


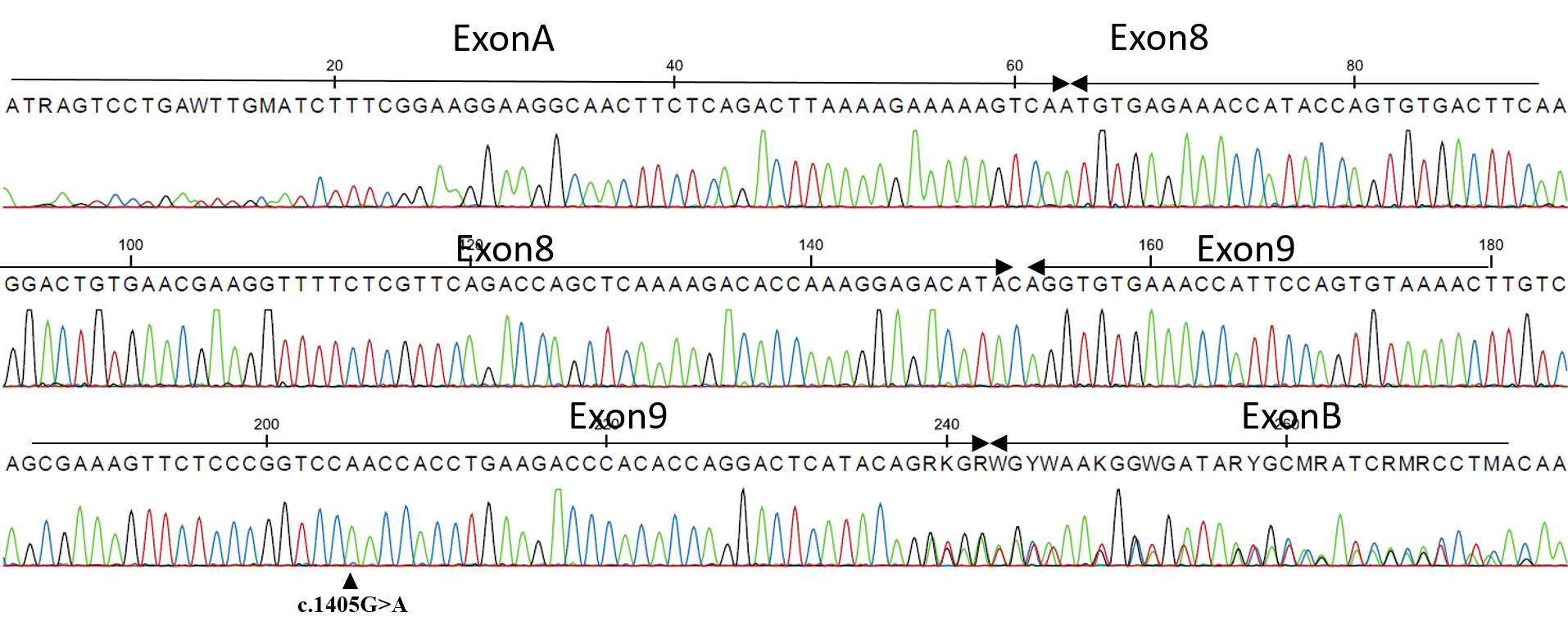


(H)　No.7, c.1405G>C


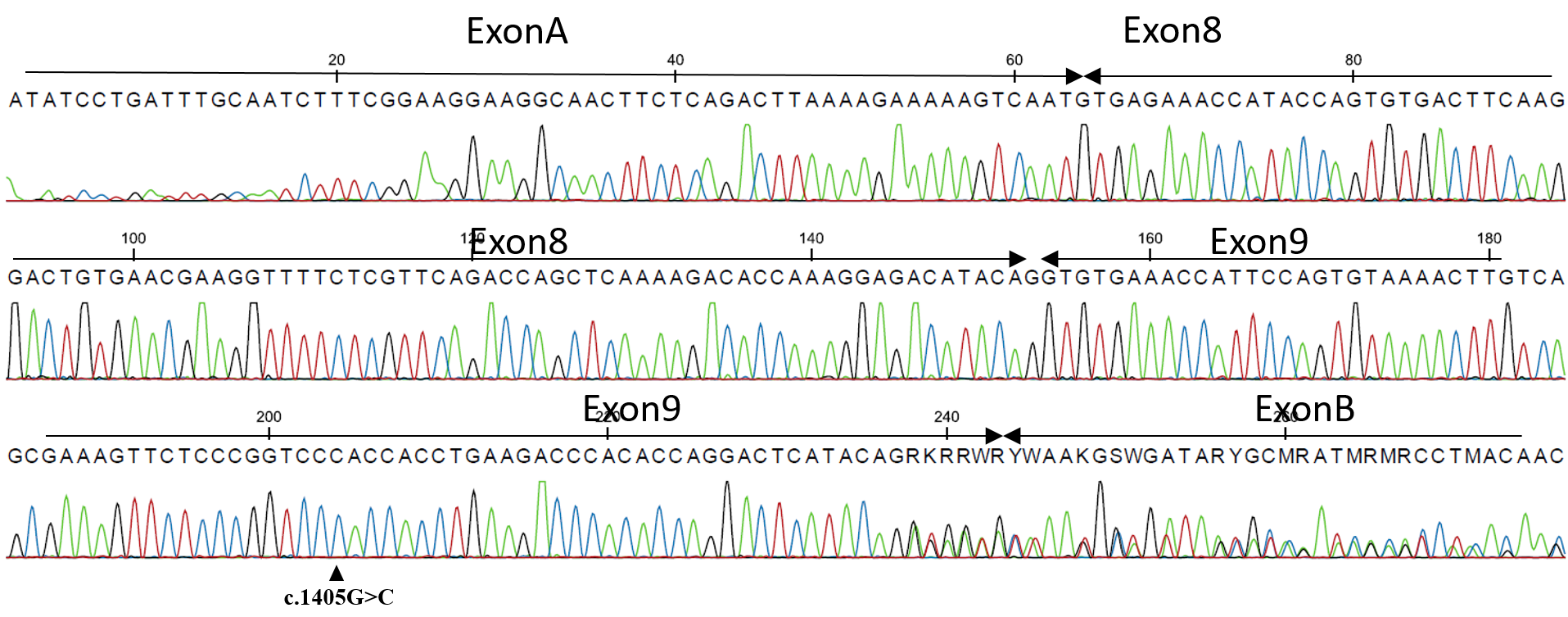


(I)　No. 8, c.1405G>T


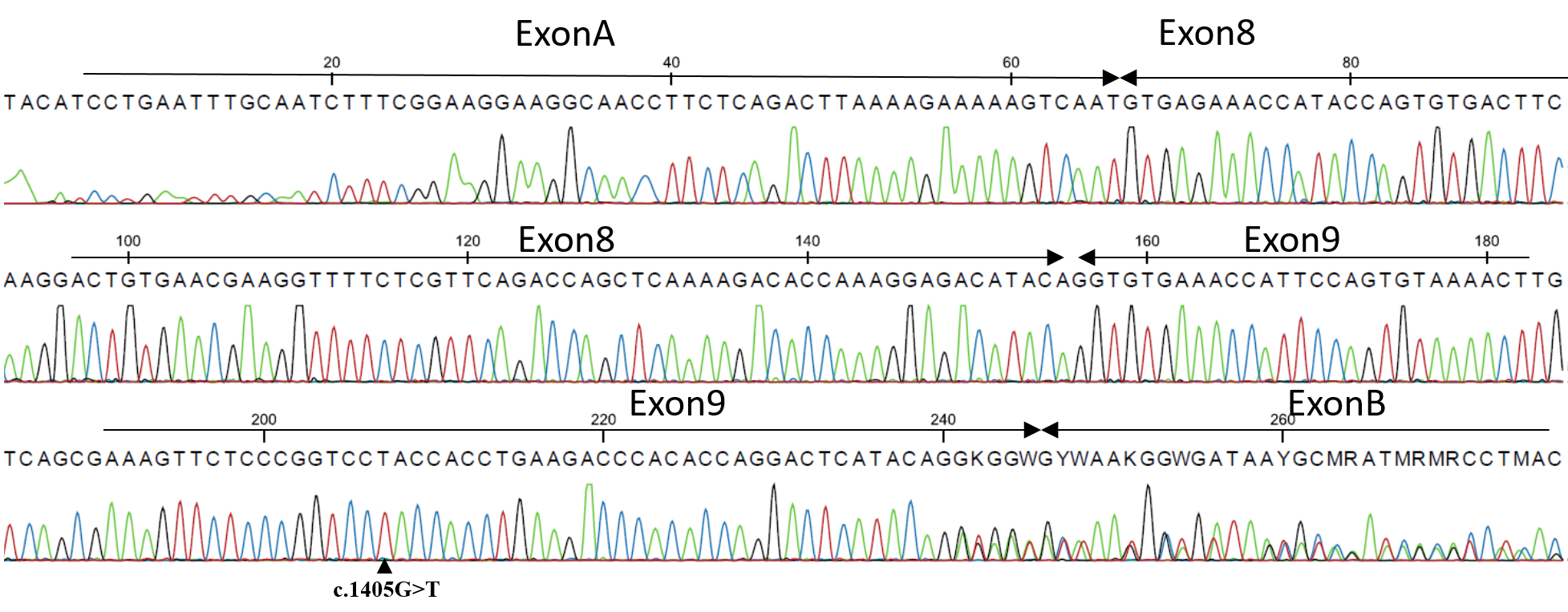


(J)　No. 9, c.1406A>G


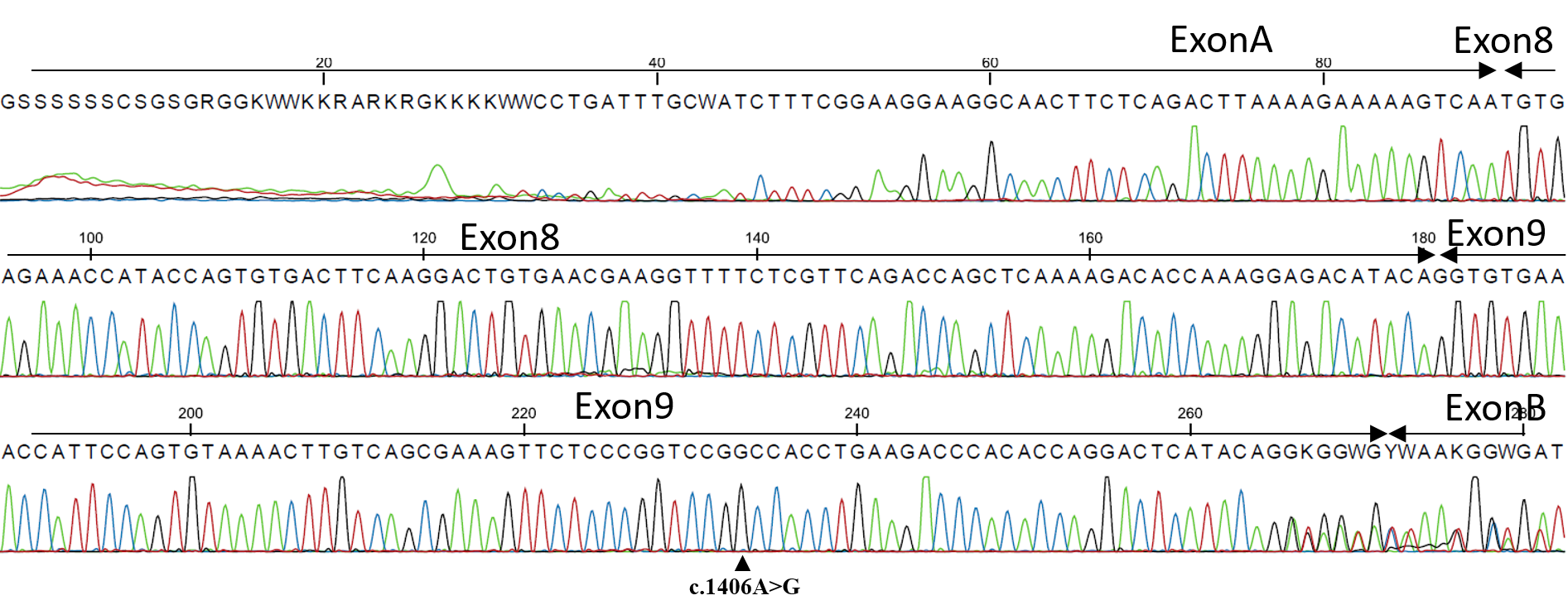


**Supplementary Figure Legends**

**Supplementary Figure S1.**

The H492v vector contains two cassette exons, A and B, separated by their natural intron into which we have inserted appropriate cloning sites. The H492 vector also contains a cytomegalovirus (CMV) enhancer-promotor and a bovine growth hormone gene (BGH) polyadenylation site.

**Supplementary Figure S2.**

(A) WT transcript exhibited exon 8 and exon 9 inclusion. (B) Transcript generated from No.1 contained exon 8 and exon 9. (C) Transcript generated from No.2 contained exon 8 and exon 9. (D) Transcript generated from No.3 contained exon 8 and exon 9. (B) Transcript generated from No.1 contained exon 8 and exon 9. (E) Transcript generated from No.4 exhibited exon 8 skipping. (F) Transcript generated from No.5 contained exon 8 and exon 9. (G) Transcript generated from No.6 contained exon 8 and exon 9. (H) Transcript generated from No.7 contained exon 8 and exon 9. (I) Transcript generated from No.8 contained exon 8 and exon 9. (J) Transcript generated from No.9 contained exon 8 and exon 9.
